# A group- and smartphone-based psychological intervention to increase and maintain physical activity in patients with musculoskeletal conditions: study protocol for a randomized controlled trial (“MoVo-App”)

**DOI:** 10.1186/s13063-020-04438-4

**Published:** 2020-06-08

**Authors:** Jiaxi Lin, Ramona Wurst, Sarah Paganini, Vivien Hohberg, Stephan Kinkel, Wiebke Göhner, Christina Ramsenthaler, Reinhard Fuchs

**Affiliations:** 1 Department of Psychiatry and Psychotherapy Medical Center, Freiburg, Germany; 2 Department of Sport Psychology, Institute of Sports and Sport Science, University of Freiburg, Schwarzwaldstrasse 175, 79117 Freiburg, Germany; 3 Schwarzwaldkliniken Bad Krozingen, Bad Krozingen, Germany; 4 Department of Health Psychology, Catholic University of Applied Sciences, Freiburg, Germany

**Keywords:** Blended intervention, App, Motivation and volition, Psychological intervention, Long term, Exercise, Sport activity, Orthopedic rehabilitation, Randomized controlled trial

## Abstract

**Abstract:**

**Background:**

Interventions designed to increase the level of physical activity are crucial in the treatment of patients with musculoskeletal conditions. The psychological group-based intervention MoVo-LISA based on the Motivation-Volition (MoVo) Process Model has been shown to effectively promote physical activity. The aim of this study is to evaluate whether a MoVo-based app (MoVo-App) subsequent to MoVo-LISA during orthopedic inpatient care can support people to increase and maintain their amount of physical activity.

**Methods/design:**

In this parallel-group randomized controlled trial, patients with musculoskeletal disorders will be randomized to either (a) a combination of the group-based intervention program MoVo-LISA to promote physical activity plus the MoVo-App or (b) the group-based intervention program alone without the app. The intervention group will receive the MoVo-App after discharge from inpatient rehabilitation. They receive help to increase and maintain their level of physical activity (initiated by the group program) by tracking their health goals, activity plans, major barriers, and barrier management that were developed during the group-based program. We will recruit 224 initially minimally active participants during orthopedic rehabilitation care. Outcomes are assessed at clinic admission; discharge; 6 weeks; and 3 (post-treatment), 6, and 12 months after discharge (follow-up). The primary outcome is sport activity (active/inactive and minutes of activity) at 6-month follow-up. Secondary outcomes are movement activity, cognitive mediators of behavioral change (e.g., self-efficacy, action planning), and health-related variables (e.g., pain intensity, depression). To evaluate intervention effects, linear mixed effects models (both on intention-to-treat basis with an additional per-protocol analysis) will be conducted with each outcome variable and with time as the within-subjects factor and group as the between-subjects factor, along with all two-way interactions and accounting for covariates as fixed effects.

**Discussion:**

This is the first evaluation of the effectiveness of an app in combination with a face-to-face group intervention to promote physical activity. The approach of using an app in addition to an effective face-to-face intervention program, both based on the MoVo model, might sustain positive intervention effects introduced in routine health care.

**Trial registration:**

The trial “A group- and smartphone-based psychological intervention to increase physical activity in patients with musculoskeletal conditions: A randomized controlled trial” is registered at the World Health Organization International Clinical Trials Registry Platform via the German Clinical Studies Trial Register (DRKS), DRKS00014814. Registered on 18 October 2018; URL: https://www.drks.de/drks_web/navigate.do?navigationId=trial.HTML&TRIAL_ID=DRKS00014814.

## Background

Musculoskeletal disorders such as chronic back pain, rheumatoid arthritis, and osteoporosis are highly prevalent conditions. The global prevalence of low back pain lasting longer than 1 month is estimated to be 23.2% [1]. The 1-year prevalence of back pain in Europe is 46.1%, and for neck and upper limb pain, it is 44.6% [2]. Furthermore, approximately 54 million (22.7%) of adults aged ≥18 years were diagnosed with arthritis in the United States between 2010 and 2012 [3]. In Germany, the prevalence rates of back pain and osteoarthritis are 22.5% and 20.6%, respectively, in people aged 20–75 [4]. Impairments of the musculoskeletal system cause high physical and economic burden to the individual and society [5, 6]. Low back pain, one of the most prevalent chronic musculoskeletal conditions, is the leading cause of years lived with disability worldwide, with other musculoskeletal conditions among the top ten causes [7].

Interventions to increase and maintain physical activity are among the most effective interventions for preventing further disease symptoms such as pain and dysfunction, and promoting health-related quality of life in patients with musculoskeletal disorders [5, 8–10]. An overview of systematic reviews found that exercise therapy yielded standardized mean differences between 0.30 and 0.65 for improving pain and function in individuals with different musculoskeletal conditions [5]. However, few patients manage to establish a stable level of physical activity behavior after inpatient treatment under their own volition [11, 12]. The Motivation-Volition concept for promoting Lifestyle-Integrated Sport Activity (MoVo-LISA; www.movo-konzept.de), a group-based intervention administered during inpatient rehabilitation, was developed to overcome low exercise adherence and to effectively promote long-term physical activity [13, 14]. Several randomized controlled trials (RCTs) (and quasi-experimental studies) have shown that MoVo-LISA has beneficial short- and long-term effects in patients with different conditions [15–19]. Promoting physical activity has consistently been shown to lead to a significant increase in exercise duration compared with preintervention or control group in psychosomatic [15], orthopedic [16, 17, 20], and cardiovascular [19] patients and in overweight or obese individuals [18]. In orthopedic patients, Fuchs and colleagues [16] reported the MoVo-LISA group as being more active than the usual care group by 28.5 min per week, even 12 months after discharge (per-protocol analyses). Moreover, investigations of treatment mediators strongly support the theoretical framework of the MoVo concept because behavior changes were shown to be based on changes in the underlying psychological factors [17, 21].

In previous studies, exercise duration measured after completion of MoVo-LISA was, on average, 156 min/week in the intervention group (IG). However, exercise duration decreased to 92 min/week at 6 months after discharge [13, 17]. New effective and cost-effective ways to prevent this decrease are needed. One way to do so could be to use technologies such as mobile applications (apps) to support participants in maintaining MoVo-LISA promoted habits and thus their level of physical activity. Several studies and systematic reviews with and without meta-analyses have shown that internet- and mobile app–based interventions can effectively and potentially cost-effectively increase and help maintain the amount of physical activity [12, 22–29]. A recent systematic review reported significant health improvements of app-based interventions targeting at physical activity in 14 of 21 studies [28].

Despite significant improvements, the positive effects of mobile apps have not been consistently reported, particularly in studies that use apps as stand-alone interventions. This might be due to internet- and mobile app–based interventions battling with repeatedly high dropout rates [30–35]. In internet-delivered physical activity behavior change programs, the average cross-study attrition rate was 23% in the IGs (reported in 28 of 34 studies) [29]. This effect seems to be more pronounced in studies using apps as stand-alone interventions. There is also evidence that multicomponent interventions lead to higher efficacy in behavioral and health outcomes than interventions consisting of apps alone [28]. Thus, blended interventions combining both face-to-face sessions and mobile material might be less prone to suffer from high withdrawal. Another advantage of blended interventions is that they can increase therapy intensity, which in turn might increase intervention effects [36]. In a systematic review of blended face-to-face and internet-based interventions for the treatment of mental disorders in adults, Erbe and colleagues found that these interventions can be more effective than no treatment [37]. Thus, an app embedded within a face-to-face intervention might help maintain the increased amount of physical activity that was acquired in a face-to-face intervention. Therefore, we developed a smartphone app based on the MoVo concept (“MoVo-App”) and adapted it to the MoVo-LISA group intervention.

The following is the primary research question:

1. Can the combination of the face-to-face group-based intervention MoVo-LISA and MoVo-App increase maintenance of the initiated physical activity level in patients with musculoskeletal disorders more than MoVo-LISA without subsequent MoVo-App use?

We hypothesize that the additional MoVo-App can help to maintain the initiated level of physical activity (by the face-to-face group program) 6 months after clinic discharge in patients with musculoskeletal disorders more than MoVo-LISA without the app.

The following are secondary research questions:

2. Can the combination of MoVo-LISA and MoVo-App improve cognitive mediators of behavioral change (e.g., self-efficacy or action planning), and health-related variables (e.g., pain intensity, depression) in patients with musculoskeletal disorders more than the same face-to-face group-based intervention without subsequent MoVo-App use?
3. Which cognitive and sociodemographic factors moderate and mediate the effects of MoVo-LISA and MoVo-App?

## Methods/design

### Study design

**Fig. 1:**
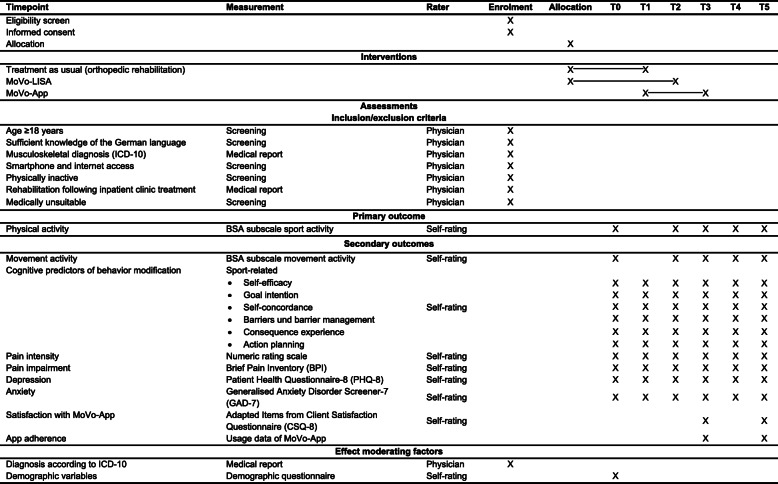
Schedule of enrollment, interventions, and assessments

This study was registered on the World Health Organization International Clinical Trials Registry Platform via the German Clinical Studies Trial Register on 18 October 2018 (DRKS00014814). For an overview of the study schedule, enrollment, interventions, and assessments, *see* the Standard Protocol Items: Recommendations for Interventional Trials (SPIRIT) [38] schedule in Fig. 1. The SPIRIT checklist [38] can be found in Additional file 1.

This is a multicenter, pragmatic RCT of parallel design with an allocation ratio of 1:1. In this RCT, we aim to evaluate whether the MoVo-App in combination with the group-based face-to-face intervention MoVo-LISA during orthopedic rehabilitation can effectively support inactive individuals to initiate physical activity, to increase their amount of physical activity (minutes per week), and to maintain this level of physical activity (physically active: yes/no and minutes per week) in the long term.

**Fig. 2:**
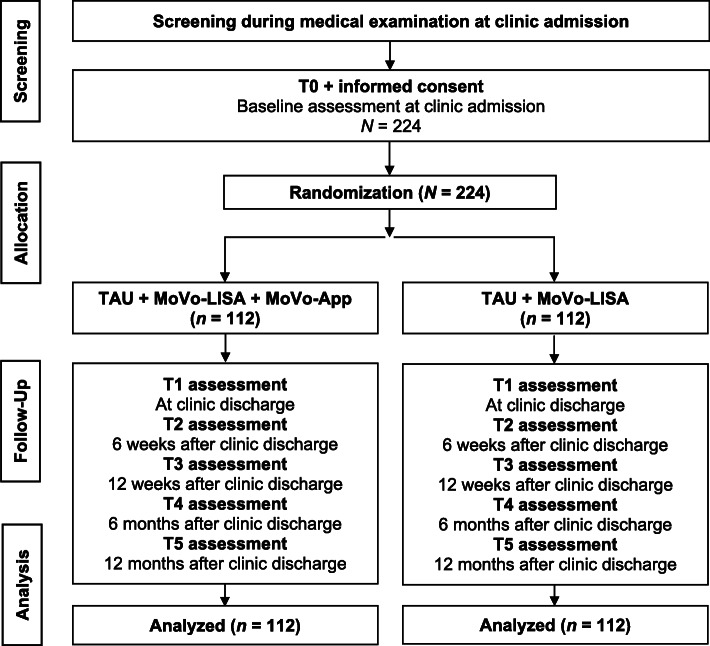
Consolidated Standards of Reporting Trials (CONSORT) flowchart

All participants receive treatment as usual (TAU) and the face-to-face group-based intervention MoVo-LISA during their clinic stay (*see* Fig. 1 and the Consolidated Standards of Reporting Trials (CONSORT) flowchart in Fig. 2). After participating in the face-to-face MoVo-LISA program in the clinic, the IG receives the MoVo-App at clinic discharge as a blended intervention. Following eligibility screening by the clinic staff, participants give informed consent and complete baseline outcome assessments prior to randomization and allocation (T0). Assessments are repeated at clinic discharge (T1), at 6 weeks (T2), and at 4 months after discharge (after the end of the MoVo-App and thus post-treatment) (T3), as well as 6 (T4) and 12 months (T5) after discharge (follow-up). All assessments are conducted through online questionnaires. The first two assessments (T0 and T1) take place in the clinic, and the participants are supported by a study assistant. All further online questionnaires are sent to the participants via e-mail. If the participants do not complete the questionnaires, they will be reminded after 2, 4, 6, and about 14 days after receiving the questionnaire. The first and second reminders are sent to the participants by e-mail. The third reminder is carried out via telephone. If the patients cannot be reached by telephone, they are called a second time.

We are conducting and reporting the RCT in accordance with the CONSORT 2010 statement [39], the supplement of the CONSORT statement for pragmatic effectiveness trials [40], and current guidelines for executing and reporting eHealth research [41]. All procedures are approved by the ethics committee of the Albert Ludwig University of Freiburg (no. 270/18).

In a pilot study from October until December 2018, the procedures for recruitment and intervention delivery in the clinics were tested and refined. During this time, 34 participants were recruited. On the basis of experiences and feedback of patients as well as clinic staff regarding conduction and implementation of the study, some adjustments and improvements were made to optimize the recruitment strategies, outcome assessments, and the usability and installation of the MoVo-App.

### Proposed sample size/power calculations

In a previous group-based MoVo-LISA trial with orthopedic patients, exercise duration was 156 min/week immediately after the intervention, which decreased to 92 min/week at 6 months after discharge [16]. If the MoVo-App is able to stabilize the post-treatment effect, a medium effect size of (standardized mean difference) *d* = 0.62 for the main effect and a small to medium effect size for the interaction effect group (MoVo-LISA + MoVo-App) × time of *d* = 0.35 at the 6-month follow-up (T4) can be expected. However, the effect size estimate for the interaction effect could only be extrapolated from interaction effects observed for group trials comparing MoVo-LISA with a wait-list control group [19]. We assume a baseline measure of the outcome to be used as a covariate and a correlation between pre- and postmeasures of 0.33 based on prior data. With a power of 80%, a significance level of 5%, and small to moderate effect sizes, and considering an estimated dropout rate of 15%, a total sample size of 224 is required.

### Recruitment

Recruitment started in January 2019 and ended in December 2019. Although this study protocol was submitted for publication after the start of recruitment (due to usual writing, commenting, and approval procedures involving all coauthors), clinical trial registration took place prior to recruitment start. Participants were recruited in two orthopedic rehabilitation clinics located in southwestern Germany. In Germany, patients with severe injuries and chronic musculoskeletal conditions have the opportunity to receive rehabilitation therapies for 3 or more weeks in an orthopedic inpatient rehabilitation clinic covered by insurance companies. The target population in this study consists of persons with musculoskeletal conditions (e.g., arthritis, chronic back pain). Participants did not receive compensation for their participation. All consecutive patients are screened by a physician for eligibility upon arrival during their first medical examination. The first participant was enrolled on 7 January 2019, and the last patient was enrolled on 3 December 2019.

### Inclusion criteria

Patients are included if they fulfill the following inclusion criteria: (1) age 18 years or older; (2) musculoskeletal diagnosis classified in chapter M of the International Statistical Classification of Diseases and Related Health Problems, 10th Revision (ICD-10) [42], diagnosed by a physician; (3) sufficient knowledge of the German language; (4) ownership of a smartphone and internet access for online assessments; and (5) being physically inactive (sport activity < 30 min/week) in the last 3 months. Exclusion criteria are (1) rehabilitation following inpatient clinic treatment (e.g., due to surgery) and (2) not able to exercise for medical reasons. Eligible patients receive an invitation to participate in the study, an information letter/leaflet, and the informed consent form and complete the baseline online questionnaire (T0) during a study information meeting with a study assistant.

### Randomization

Randomization and allocation of participants to the two groups were done in advance by an independent researcher (CR) by means of a web-based program (https://www.sealedenvelope.com/) using permuted block randomization with variable block sizes of 2, 4, and 6 (randomly arranged) and a ratio of 1:1. Participants are stratified by center. The centers involved are two orthopedic clinics in southwestern Germany where recruitment is conducted consecutively. Participants are given randomly generated treatment allocations within sealed opaque envelopes. Once a participant is consented to participate in the study, the envelope is opened, and the patient is then offered the allocated treatment regimen. An independent researcher (CR) prepares the envelopes and is responsible for the allocation of participants to trial arms.

### Content of the face-to-face group intervention MoVo-LISA

The psychological group program MoVo-LISA (Motivation-Volition concept to support Lifestyle-Integrated Sports Activity; three to eight patients per group) is based on both motivation theories of health behavior [43–45] and volition theories of action planning and action control [46–48]. The theoretical framework underlying the MoVo-LISA intervention program is the MoVo process model. Its core idea is the differentiation between motivational and volitional strategies [21]. Motivational strategies are used to help form a strong and self-concordant goal intention. Volitional strategies are needed to implement competencies and action control [13]. MoVo-LISA is conceptualized as a short and economical psychological program based on a standardized curriculum. It has been developed specifically for an inpatient rehabilitation setting and has been shown to be effective in orthopedic patients [16, 17, 20]. In this trial, MoVo-LISA is offered to all randomized participants in both the intervention and control groups by the physiotherapists or sport and exercise therapists in each clinic. Before recruitment, all MoVo-LISA instructors were trained in the standardized program by the scientific project team during a 2-day course. In addition, a kickoff meeting was organized for all clinic staff in order to inform them about the study and to familiarize them with the MoVo concept.

The program MoVo-LISA consists of two group sessions and one individual session in which patients learn how to integrate physical activity into their daily lives. It does not consist of actual physical exercise but focuses on teaching the patients how to get and stay physically active after inpatient rehabilitation. The patients prepare specific activity plans for implementation in their everyday lives based on their individual needs and living context. Hence, the activity plans of the participants vary highly from aerobics to stretching and stabilizing exercises and are based on the recommendations that were developed during their orthopedic rehabilitation. In addition, they identify barriers that discourage them from being active and develop individual strategies to cope with those barriers. After discharge from the clinic, participants receive a postal reminder and a telephone interview. A detailed description is given by Fuchs and Göhner [16, 49]. The five main components of MoVo-LISA are listed in Table 1.

**Table 1:** Elements of the MoVo-LISA program

Components	Time of event	Duration	Content/aim
First group meeting	Days 1–3 after clinic admission	60 min	To reflect on health goals and ideas for physical activity
Homework	After first group meeting	–	To prepare an individual activity plan
One-to-one interview	1 week after first group meeting	10 min	To refine the individual activity plan
Second group meeting	1–3 days before clinic discharge	90 min	To discuss barriers and barrier management to establish an activity protocol
Postal reminder	3 weeks after discharge		To remind patients of the two activity plans and the importance of their individual barrier management
Telephone call	5–6 weeks after discharge	10 min	To check the implementation of the activity plans and barrier management and to offer support in case of problems

### MoVo-App

The MoVo-App is based on the MoVo process model and was developed by a multidisciplinary team of psychologists, sport scientists, and computer scientists from the University of Freiburg. The aim was to develop an app that can be provided to individuals after clinic discharge subsequent to the group-based face-to-face intervention MoVo-LISA. The elements of the program are implemented within the MoVo-App to support participants in maintaining their acquired strategies and to put their activity plans into action within the upcoming 12 weeks after clinic discharge. We developed the MoVo-App for smartphones running iOS or Android operating systems.

After the installation of the MoVo-App, participants in the IG receive help with entering their health goals, activity plans, major barriers, and barrier management strategies developed during the MoVo-LISA group program into the app. Over the course of 12 weeks after discharge, the MoVo-App sends regular reminders to participants regarding their activity plan prior to the scheduled activity. Participants can record on the same day of the activity plan what percentage of their plan they have achieved in order to track their progress. Each week, participants are asked to evaluate their performance in terms of their satisfaction regarding the completion of their activity plans for that week. Participants can make adjustments to their activity plans or barrier management strategies if needed. All usage data of the app will be saved in the back end of the smartphone. At the end of the 12-week period, all users will be asked to finish the intervention and upload their usage data to a sharing platform (back4app.com) via a shortcut in the app. In addition to receiving the MoVo-App, participants in the IG have unrestricted access to TAU.

### Control condition

The control group receives MoVo-LISA during the clinic stay and has unrestricted access to TAU.

### Treatment as usual

TAU consists of a 3-week complex interdisciplinary and multimodal rehabilitation schedule. It comprises medical, physical, and psychological therapies specifically tailored to orthopedic patients. (More information on the German system of medical inpatient rehabilitation is given by Jäckel and colleagues [50]). Because TAU after clinic discharge may vary widely, TAU in this time will not follow a specific protocol.

### Assessments

The primary outcome is the level of sport activity that is assessed using the validated Movement and Sport Activity Questionnaire (BSA-F [51]). Secondary outcomes are movement activity, cognitive predictors of behavioral change, and health-related variables. According to the MoVo process model, it is necessary to change the underlying cognitions and self-regulatory skills in order to attain long-term behavior modification. The cognitive variables postulated by the MoVo process model assessed in this trial are sport-related self-efficacy, goal intention, self-concordance, action planning, perceived barriers, and barrier management, as well as sport-related consequence experiences. Experience of pain is considered a major health indicator among orthopedic patients. On the basis of the recommendations of the Initiative on Methods, Measurement, and Pain Assessment in Clinical Trials [52, 53], we selected pain-related outcome measures, including physical and emotional functioning. Demographic and clinical variables (e.g., age, sex, ICD-10 diagnosis), intervention adherence, and satisfaction are also assessed. The outcome measures and time points of interest are summarized in Fig. 1.

### Primary outcome

The level of sport activity in minutes per week is measured with the BSA-F [51]. This is a validated German self-report instrument used to measure the level of movement activities (functional physical activities of daily living; *see* “Secondary outcomes” section below) and sport activities (sport- and health-related exercises, such as soccer, jogging, Nordic walking, Pilates, or fitness training) in minutes per week. For the sport activity subscale, the participants list a maximum of three sport activities in which they have engaged within the last 4 weeks. For each activity episode, participants indicate the frequency and duration in minutes. Activities that do not target larger groups of skeletal muscles and do not lead to maintenance of or increases in endurance, power, coordination, or flexibility are classified as invalid activities for this group. For each valid activity, an activity amount in minutes per week is calculated by multiplying frequency and duration divided by 4. All single amounts of the named sport activities are added to obtain the individual’s score on the Sport Activity Index. The validity of the BSA-F has been shown in several studies. However, data do not permit an analysis of the reliability of the instrument [51].

### Secondary outcomes

#### Movement activity

Movement activity is measured with the BSA-F using the respective subscale [51]. The movement activity subscale consists of work- and leisure time–related movement activities within the last 4 weeks. Participants first rate their activity levels during work with regard to the frequency of (1) sitting (inversely coded), (2) moderate movement, and (3) intensive movement on a scale from 0 = not at all to 3 = a lot. Participants are then asked to indicate the duration in minutes per day and the frequency during the last 4 weeks for the eight most common movement activities in everyday life (e.g., riding a bike to work or carrying home groceries by foot). For each activity, an amount in minutes per week is calculated by multiplying frequency and duration divided by 4. These are added to obtain the individual’s score on the Movement Activity Index for the work- and leisure time–related movement activities separately.

#### Self-efficacy

In accordance with Schwarzer and colleagues [54–56], three different types of self-efficacy will be assessed: the belief that someone is able to (1) begin regular physical exercise, (2) maintain regular activity over a longer time period, and (3) resume regular activity after interruption. Each type of self-efficacy is measured with one item; the scores of the three items will be combined into one mean value. The response format is a 6-point Likert scale ranging from 0 = I don’t feel capable at all to 5 = I feel 100 per cent capable. An earlier study [15] yielded a satisfactory internal consistency ranging from α = 0.75 to α = 0.92 at different time points.

#### Goal intention

The strength of goal intention is assessed with a single item: “How strong is your intention to exercise regularly within the following weeks and months?” Participants can respond on a 6-point Likert scale ranging from 0 = I do not intent to exercise regularly to 5 = I do have a strong intention to exercise regularly.

#### Self-concordance

Self-concordance is measured using the German SSK scale (Sport-und bewegungsbezogene Selbstkonkordanz) [57], consisting of 12 items based on the self-concordance model of Sheldon and Elliot [45]. The SSK scale involves four subscales that measure the intrinsic, identified, introjected, and extrinsic motivation for being physically active. Only participants who indicated having at least a weak exercise-related goal intention (strength of goal intention > 1) are receiving this questionnaire. The 4-point Likert scale ranges from 1 = not true to 4 = true.

This instrument has proved to be a reliable and valid measure of exercise-related self-concordance [58]. In two separate studies reported by Seelig and Fuchs [58], the reliability of the subscales ranged from α = 0.70 (identified motivation) to α = 0.82 (intrinsic motivation). Evidence of validity was provided by a correlation of *r* = 0.47 between the SSK scale and the intention to engage in physical exercise [58].

#### Barriers and barrier management

The two scales of sport-related situational barriers and sport-related barrier management [59] allow detailed analyses of the process of volitional action control in the field of sport and physical activity. The questionnaire to assess perceived barriers contains 13 items presenting the most common sport-related situational barriers. Participants are asked how often these barriers discourage them from engaging in sport activity. The questionnaire assessing strategies for barrier management contains 15 items assessing the most common barrier management strategies. In this questionnaire, participants are asked how often they use these strategies. In both questionnaires, participants rate the items on a 4-point Likert scale ranging from 1 = almost never to 4 = almost always. Both scales are reliable (sport-related barrier management: α = 0.78, situational barriers: α = 0.81) and valid instruments [59].

#### Consequence experience

Expectation about sport-related consequence experience is assessed using an instrument developed and validated by Fuchs [60]. It includes 16 positive and negative versions of outcome experience of physical exercise. Participants are asked to respond on a 4-point Likert scale ranging from 1 = not true to 4 = true. The positive and negative experience items are summed separately. The difference of positive–negative items is derived to provide an outcome experience index [60].

#### Action planning

Participants are asked whether they already know which physical exercise to perform in the following weeks and months. If the answer is “yes,” patients will be asked to note this activity. An opportunity is provided to name a second activity. For each of these activities, participants are asked whether they already know (1) when and (2) where they will perform it, (3) how they will get there, and (4) how often and (5) with whom they will perform the activity. An action planning score is formed by summing the number of positive answers (including naming the activity plus planning details).

#### Pain intensity

We are using an 11-point numerical rating scales for rating the worst, least, and average pain during the last week as well as the current pain level, and the mean of the four scales is calculated. The participants evaluate their pain from 0 to 10, with 0 meaning “no pain” and 10 meaning “pain as bad as you can imagine.” Dworkin and colleagues reported the internal consistency of a composite pain score averaging ratings of current pain, average pain, and worst pain as satisfactory to high (α = 0.77). The composite score was normally distributed, contrary to the individual ratings [61].

#### Pain interference

The Brief Pain Inventory (BPI) allows participants to rate the degree to which their pain interferes with common aspects of emotional and physical function [62, 63]. The seven BPI interference items assess pain interference during the past 24 h on an 11-point scale from 0 (“does not interfere”) to 10 (“completely interferes”). The BPI is a reliable (α = 0.88) and valid measure of the interference of pain with physical and emotional functioning [64].

#### Depression

The eight-item Patient Health Questionnaire depression scale [65] is a valid diagnostic measure of the severity of depressive symptoms based on the *Diagnostic and Statistical Manual of Mental Disorders, Fifth Edition* (DSM-5). With the exception of the symptom of suicidal ideation or self-injurious thoughts, each of the nine DSM-5 criteria for major depression can be scored from 0 (“not at all”) to 3 (“nearly every day”) in the past 2 weeks. The instruments shows satisfactory validity and reliability [66].

#### Anxiety

The seven-item Generalized Anxiety Disorder scale [67] describes the most prominent diagnostic features of the DSM-5 criteria A, B, and C for generalized anxiety disorder. These can be scored from 0 (“not at all”) to 3 (“more than half the days”) during the last 2 weeks. Its reliability and validity is good [65] with excellent internal consistency (α = 0.92) and good test–retest reliability (intraclass correlation coefficient, 0.83).

#### Intervention adherence

The attrition rate (percentage of individuals who no longer use the app, as assessed by their log-on data) gives an estimate of the individuals’ intervention adherence. Also, usage time will be assessed for every user who will upload the app at the end of the intervention.

#### Patient satisfaction

The Client Satisfaction Questionnaire [68, 69] has been optimized for the assessment of client satisfaction with internet interventions (Client Satisfaction Questionnaire adapted to Internet-based interventions [CSQ-I] [70]). We adapted this measure for the use of apps by replacing the word “training” with “app.” The CSQ-I is a valid and reliable (α between 0.93 and 0.95) measure of client satisfaction [70].

### Statistical analyses

The primary outcome of physical activity assessed with the respective subscale of the BSA-F at T4 (post-treatment of MoVo-App) will be analyzed as a dependent variable and its baseline value as a covariate, adjusted for sex and age and potentially other covariates. Continuous secondary outcomes will be analyzed accordingly. The statistical analysis for all research questions will be based on linear mixed-effects models with restricted maximum likelihood estimation to account for missing data [71]. The model is a mixture model, thus assuming two distinct distributions: (1) a binomial model (patients becoming physically active from inactivity and vice versa) and (2) a Gaussian distribution of minutes of physical activity per week for those being physically active. In the linear mixed-effects models, time (T1 to T5) will be the within-subjects factor, and intervention (MoVo-LISA and TAU plus MoVo-App vs. MoVo-LISA and TAU) will be the between-subjects factor. All two-way interactions will be considered. Main effects and interactions will be estimated for time points T3 to T5.

Baseline minutes of sport activity, age, sex, and body mass index will be included as covariates, along with other relevant demographic and clinical characteristics (e.g., disease, pain intensity, pain interference) collected through patient self-report. The model will allow for random intercepts, and all other factors will be treated as fixed effects. All statistical analyses will be performed using R software [72].

We expect to see a skewed distribution in our primary outcome physical activity, and, following guidance [72, 73], we will use transformations or consider using generalized or mixture-distribution mixed models [72]. Effects will be considered significant if *p* < 0.05. Assumptions for general, generalized, or mixture-distribution models will be checked graphically and with tests appropriate to the model [74, 75]. In addition, we will run several model checks by comparing increasingly complex models with different covariance structures, link functions, and estimation procedures with a null model via likelihood ratio tests and comparison of fit via the Akaike information criterion and Schwartz’s Bayesian information criterion [76].

To assess the mediating and moderating effects of psychosocial factors, the linear mixed-effects model of repeated measures will be extended by including psychosocial factors as random effect covariates. Their added effect as covariates, their interaction with IG, and a three-way interaction with IG and time will be estimated. It will be considered whether and how psychosocial intermediary effects depend on the intervention. Effects will be considered significant if *p* < 0.05.

The analysis does not consider a cluster effect of using two sites for the intervention. This decision is based on the high standardization in the delivery of the intervention and all study procedures in both sites. Differences in patient population between clinics will be accounted for by including demographic and clinical variables as covariates in the analysis. Furthermore, clinic was also used as a stratification factor in the randomization scheme (as detailed above).

Standardized mean differences and 95% confidence intervals will be calculated to measure the between-group effect size at post-treatment (T3) and follow-up (T4, T5). All analyses will be performed on an intention-to-treat basis. Completer (per-protocol) analyses will be conducted to investigate the influence of study attrition on study results. In addition, we will analyze the extent to which participants adhere to physical activity in accordance with the National Recommendations for Physical Activity and Physical Activity Promotion [77].

### Ethical considerations

Participants eligible for the study receive detailed study information and are asked for their participation (informed consent). Smartphone-based interventions have proved to be effective in a variety of RCTs without clinically meaningful side effects being reported yet [28, 78]. Due to the focus on patients with musculoskeletal conditions, the participants are at an increased risk of injury compared with the general population. The medical suitability of participants for the intervention is recorded continuously as part of the inpatient rehabilitation diagnostic assessment and treatment. Data security and confidentiality are guaranteed under the General Data Protection Regulation Act. A data security concept is created together with the data security department at the University of Freiburg, Germany. Central ethical approval has been confirmed from the Albert Ludwig University of Freiburg (DRKS00014814).

### Source of monetary or material support

The study is supported via the institutional budget with no external funding. There are no sponsors involved in this study.

## Discussion

The present trial focuses on individuals with musculoskeletal conditions. The purpose of the MoVo-App trial is to evaluate a blended intervention of inpatient, face-to-face treatment coupled with a mobile app to help initiate and promote the maintenance of physical activity in this vulnerable patient group. Previous studies have shown that the initial adoption of physical activity is possible and successful in this population. Most studies to date have not succeeded in implementing long-term habit change in participants.

In a rigorous evaluation, this trial addresses some of the gaps of prior studies. First, given that many people have busy lifestyles, they value convenient access to health behavior change programs [79]. In this context, the MoVo-App offers the opportunity to support behavioral interventions in real-life situations. Second, although apps have a high potential to support individuals to become active more sustainably, most of the apps are lacking evidence-based content [80]. In the development of the MoVo-App, we systematically translated the theoretical concepts of the MoVo process model into the app. Third, outcomes in this study not only comprise physical activity behavior but also measure health-related variables (e.g., pain intensity, depression) and underlying psychological mediators of behavior change as postulated within the MoVo concept. This will allow a deeper understanding of the treatment processes within MoVo-LISA and the MoVo-App, which in turn can help to further develop the intervention. Fourth, by implementing the intervention directly into routine healthcare, we will be able to enroll a more naturalistic sample. Most trials of internet- and mobile app–based interventions suffer from a selection bias with mostly highly educated and female participants self-selecting into these interventions [35, 81]. Due to the consecutive routine care sample, self-selection bias can be minimized in this trial. Finally, MoVo-LISA is conceptualized to be used for a wide range of settings and indications. Therefore, the blended intervention of MoVo-LISA and the MoVo-App can easily be transferred to other (healthcare) settings as well as health conditions.

Some limitations need to be addressed. First, the primary outcome is assessed with a questionnaire that is only available in German and does not provide a minimal clinically important difference. Second, there are no objective measures of the primary outcome physical activity conducted. If the results of this study indicate that the blended intervention of MoVo-LISA and MoVo-App is more effective than MoVo-LISA alone, its clinical impact on the promotion and maintenance of an increased level of physical activity could be substantial.

## Trial status

This is protocol version number 3, dated June 04, 2020. Recruitment began on 7 January 2019; the last patient was enrolled on 3 December 2019. The first results of this study are expected in 2021.

## Supplementary information

**Additional file 1.** SPIRIT 2013 Checklist: Recommended items to address in a clinical trial protocol and related documents*.

## Data Availability

The final dataset, excluding any demographic and personal information allowing identification of participants, and the statistical analysis code will be accessible from the corresponding author upon reasonable request.

## Abbreviations

BPI: Brief Pain Inventory
BSA-F: Movement and Sport Activity Questionnaire
CONSORT: Consolidated Standards of Reporting Trials
CSQ-I: Client Satisfaction Questionnaire adapted to Internet-based interventions
DRKS: German Clinical Studies Trial Register
ICD-10: International Statistical Classification of Diseases and Related Health Problems, 10th Revision
IG: Intervention group
MoVo: Motivation-Volition concept
MoVo-LISA: Motivation-Volition concept to support Lifestyle-Integrated Sports Activity
MoVo-App: MoVo-based smartphone application
RCT: Randomized controlled trial
SPIRIT: Standard Protocol Items: Recommendations for Interventional Trials
TAU: Treatment as usual
